# Prenatal diagnosis of a trisomy 7/trisomy 13 mosaicism

**DOI:** 10.1186/1755-8166-5-8

**Published:** 2012-01-27

**Authors:** Karin Huijsdens-van Amsterdam, Daniela QCM Barge-Schaapveld, Inge B Mathijssen, Mariëlle Alders, Eva Pajkrt, Alida C Knegt

**Affiliations:** 1 Department of Clinical Genetics, Academic Medical Center, Amsterdam, The Netherlands; 2 Department of Fetal Medicine, Academic Medical Center, Amsterdam

**Keywords:** double autosomal aneuploidy, mosaicism, trisomy 7, trisomy 13

## Abstract

Double aneuploidy mosaicism of two different aneuploidy cell lines is rare. We describe for the first time a double trisomy mosaicism, involving chromosomes 7 and 13 in a fetus presenting with multiple congenital anomalies. No evidence for chimerism was found by DNA genotyping. The origin of both trisomies are consistent with isodisomy of maternal origin. Therefore, it is most likely that the double trisomy mosaicism arose from two independent events very early in embryonic development. The trisomy 7 and 13 cells were shown to be of maternal origin.

## Background

Double aneuploidy mosaicism of two different aneuploidy cell lines is a rare event [1]. The most frequently described combinations are a monosomy X cell line with a cell line containing a trisomy of an autosome. In literature, mosaicism of a monosomy X cell line with either a trisomy 7, 8, 10, 13, 18 or 21 have been reported [2-7]. Double autosomal trisomies are even more sporadic, and to date combinations of trisomies of the chromosomes 8 and 14, chromosomes 8 and 21, chromosomes 13 and 18, chromosomes 13 and 21, and chromosomes 18 and 21 have been reported [1,8-12].

Here, we present the first report of a double trisomy mosaicism involving chromosomes 7 and 13 in both amniotic fluid and subsequent FISH analysis of fibroblasts in a fetus presenting with multiple congenital anomalies.

## Case presentation

A 17-year-old healthy woman of a non-consanguineous couple, gravida 1, para 0, was referred at 21+0 weeks' gestation because of multiple structural anomalies. There was no history of familial congenital anomalies or drug use. The pregnancy was unplanned but welcome. A dating scan had been performed at 8 weeks' gestation. A 12 weeks' scan was not performed since the parents did not wish screening for Down syndrome. The fetus showed growth restriction with a large bilateral cleft lip and palate with severe micrognathia. The caput showed mild brachycephaly, with an enlarged cisterna magna (> p95). The left little finger was bowed and there were mild clubfeet. Following genetic counselling the patient elected to have an amniocentesis for karyotyping. Moreover, the parents requested a termination of pregnancy.

Labour was induced at 22+2 weeks and a female baby was born, who died shortly after birth. Her birth weight was 370 g. Autopsy confirmed the bilateral cleft lip and palate as well as the retrognathia, and revealed an internal malrotation of the digestive tract with the ileocecal valve situated in the upper abdomen on the left side and bicornate uterus. All body and organ measures were within normal range for the given gestational age. X-ray showed no developmental anomalies of the skeletal system. Brain autopsy found no cerebral developmental anomalies, except for microglial upregulation, most likely as a result of hypoxic damage. Finally, the placenta showed no abnormalities, with the exception of a slight degeneration.

Fetal chromosome analysis of *in situ *cultured amniocytes revealed a double trisomy mosaicism: mos 47, XX, +7[6]/47, XX, +13[18]. A trisomy 13 was seen in 75% of the amniocytes (18/24 metaphases) and in three different cultures. The remaining 25% of the cells (6/24 metaphases) showed a trisomy 7, also in two different cultures. There was no cell line with a normal karyotype, nor did we detect cells with a trisomy for both chromosome 13 and 7.

**Figure 1:**
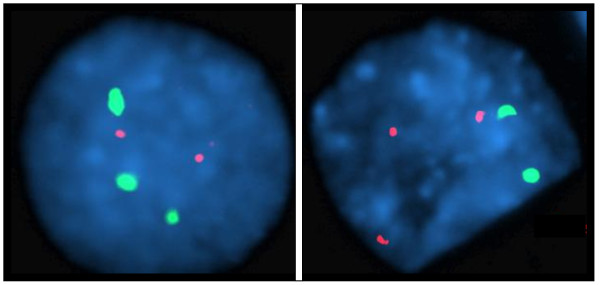
**Interphase FISH results**. FISH was performed with probes for chromosome 7 (Abbott-Vysis CEP 7, 7p11.1-q11.1, spectrum green) and chromosome 13 (Abbott-Vysis LSI 13 RB1, 13q14.2, spectrum orange). The left panel shows a cell with a trisomy 7 and a disomy 13. The right panel displays a cell with a trisomy 13 and a disomy 7.

FISH analysis of fetal skin tissue obtained after autopsy confirmed the trisomy 7 and trisomy 13 mosaicism. FISH was performed with probes for chromosome 7 (Abbott-Vysis CEP 7, 7p11.1-q11.1) and chromosome 13 (Abbott-Vysis LSI 13 RB1, 13q14.2) (Figure 1). The hybridization pattern shows 26% trisomy 7 and disomy 13 (41/159 interphase cells). Another 70% showed disomy 7 and trisomy 13 (111/159 interphase cells). In 4% (7/159 interphase cells) a normal signal pattern was seen (disomy 7 and disomy 13). This may represent a true disomy for chromosomes 7 and 13, or a FISH artefact. There were no cells with both trisomy 7 and 13, similar to the findings in the amniotic fluid earlier.

DNA genotyping using 16 STR markers (Powerplex 16 kit Promega) showed the presence of a single maternal and a single paternal allele in the DNA extracted from amniotic fluid cells for all informative markers, without evidence for chimerism (paternal allele, 14 markers informative; maternal allele 9 markers informative), suggesting that the trisomy 7 and the trisomy 13 cell lines were not a result of two different zygotes.

**Figure 2:**
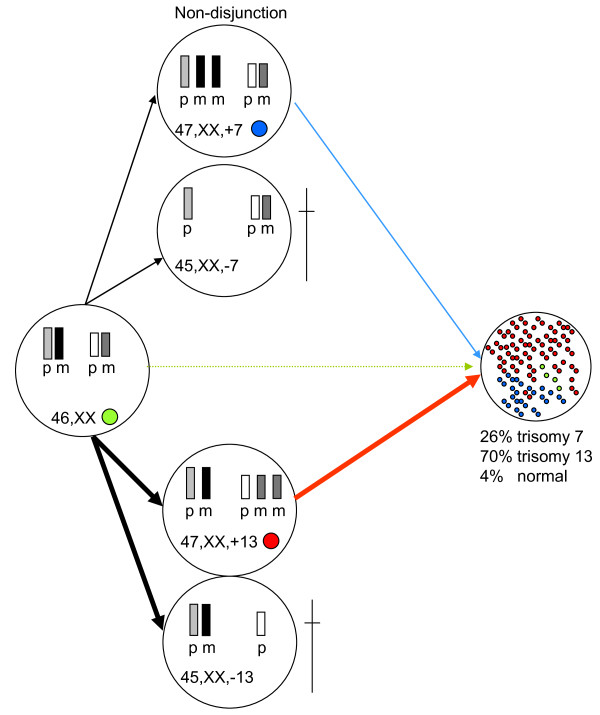
**Possible mechanism for the origin of double aneuploidy mosaicism in this fetus**. Schematic representation of the mechanisms that may have lead to a mosaic pattern of both trisomy 7 and trisomy 13 cells in a single fetus. Two independent non-disjunction events may have taken place in a 46, XX zygote, resulting in both a trisomy 7 cell line (26%) and a trisomy 13 cell line (70%). Both monosomic cells are not viable. In fetal tissue, 4% of the cells in fetal tissue showed a normal signal pattern with FISH.

Theoretically, several mechanisms may have lead to this mosaic pattern of both trisomy 7 and trisomy 13 cells in a single fetus (Figure 2). First, two independent non-disjunction events may have taken place in a 46, XX zygote. However, no cells in amniotic fluid and only 4% of cells in fetal tissue with a normal karyotype were detected. Second, two independent losses (anaphase lagging) may have occurred in a 48, XX, +7, +13 zygote. This option does not seem likely, since no cells were detected with a trisomy for both chromosome 13 and 7. A third option is a trisomic fetus with either a trisomy 7 or 13. In a part of the cells, trisomic rescue may occur, followed by a non-disjunction event, resulting in a fetus with two different trisomic cell lines.

To establish the origin of the chromosomes 7 and 13, DNA analyses was performed. Seven markers for chromosome 13 (D13S217, D13S171, D13S263, D13S159, D13S158, D13S173, D13S285) were informative and showed an increased contribution of the maternally inherited allele (confirmed by QF-PCR, Tepnel). Also, four informative chromosome 7 markers (D7S664, D7S2427, D7S2476, D7S483) showed an increased contribution of the maternally inherited allele. The results are consistent with an isodisomy 7 and an isodisomy 13 of maternal origin. Since no heterodisomic markers were seen, a postfertilization error very early in embryonic development seems most plausible. In literature, no evidence was found for higher risks for chromosomal trisomies in women under the age of 20 [13].

Mosaic trisomy 7 is not an unusual finding in chorionic villus sampling, but rarely confirmed at amniocentesis. To our knowledge, trisomy 7 mosaicism has been described in about 18 patients, half of which were regarded as clinically normal. However, in a majority of those patients, the trisomy 7 is seen in skin fibroblasts, but not in lymphocytes. Common features are pigmentary changes of the skin, facial asymmetry and short palpebral fissures [14]. Anomalies found resemble components of the VATER association [14,15]. In the current fetus, the ultrasound findings and anomalies seen at autopsy seem more in concordance with a (mosaic) trisomy 13, rather than with a (mosaic) trisomy 7 (table 1).

**Table 1:** Contribution of trisomy 7 and trisomy 13 cells to the abnormalities seen at autopsy.

Congenital abnormality	assumed to be attributed by trisomy of chromosome
bilateral cleft lip and palate	7/13

micrognathia/retrognathia	13

enlarged cisterna magna	7/13

left little finger bowed	13

mild clubfeet	7/13

malrotation of the digestive tract	13

bicornate uterus	13

## Conclusions

We describe a double aneuploid foetus with two different trisomic cell lines: one with a trisomy 7 (approximately 26%) and one with a trisomy 13 (approximately 70%). No evidence for chimerism was found by DNA genotyping. The origin of both trisomies are consistent with isodisomy of maternal origin. Therefore, it is most likely that the double aneuploidy seen arose from two independent events very early in embryonic development.

## Consent

Written consent was obtained from the parents of the patient for publication of this case report and accompanying images.

## Competing interests

The authors declare that they have no competing interests.

## Authors' contributions

KH and DB wrote the manuscript. KH performed cytogenetic analyses. DB and IB performed genetic counselling. MA carried out genotyping experiments. EP performed clinical investigation and AK coordinated the investigations.

All the authors have read and approved the final manuscript.
